# A Proposal for Neurocognitive Assessment in Spanish-Speaking Adults With Phelan-McDermid Syndrome: A Case Report

**DOI:** 10.7759/cureus.85197

**Published:** 2025-06-01

**Authors:** Yvonne Flores Medina, Aideé Gonzalez Gutierrez, Juan Jorge Palacios Casados, Mario Alberto Puente Torres, Doris Gutierrez Mora

**Affiliations:** 1 Clinical Research, Instituto Nacional de Psiquiatría Ramón de la Fuente Muñiz, Mexico City, MEX; 2 Clinical Neuropsychology, Facultad de Estudios Superiores Iztacala, Universidad Nacional Autónoma de México, Tlalnepantla de Baz, MEX; 3 Genetics, Instituto Nacional de Psiquiatría Ramón de la Fuente Muñiz, Mexico City, MEX; 4 Psychiatry, Instituto Nacional de Psiquiatría Ramón de la Fuente Muñiz, Mexico City, MEX

**Keywords:** bipolar disorder, neuropsychology, phelan-mcdermid syndrome, phonological discrimination, tactile perception

## Abstract

The main objective of this case report is to make a proposal for a specialized neurocognitive assessment battery designed to evaluate critical cognitive domains in Spanish-speaking adults with Phelan-McDermid syndrome (PMS). This battery includes measures for language processing, tactile perception, executive function, and adaptive behavior, providing a structured framework for assessing neurocognitive functioning in this population. PMS is a rare neurodevelopmental disorder caused by deletions or mutations in chromosome 22q13.33, leading to SHANK3 haploinsufficiency. This genetic alteration results in a range of clinical manifestations, including severe intellectual disability, profound language impairment, hypotonia, seizures, sensory abnormalities, and autistic traits. PMS presents significant challenges in neurocognitive assessment, particularly in Spanish-speaking adults, due to the absence of standardized diagnostic tools adapted to their needs. This case report describes a 29-year-old Spanish-speaking woman, genetically confirmed to have PMS due to a 22q13.33 deletion affecting 28 genes, including SHANK3. Her developmental history included early hypotonia, global developmental delays, limited language acquisition, and difficulties in adaptive behavior. A comprehensive neuropsychological evaluation was conducted using the Peabody Picture Vocabulary Test-III, Battelle Developmental Inventory, Behavior Rating Inventory of Executive Function-Adult Version (BRIEF-A), Adaptive Behavior Assessment System-II, Programa Integrado de Exploración Neuropsicológica Test Barcelona-2 (PIEN-2) subscales, and Ekman and Friesen Adapted Test. The results revealed severe impairments in expressive language, phonological discrimination, and tactile perception. The patient exhibited significant difficulties in recognizing emotions, particularly fear, sadness, and neutral expressions. Adaptive behavior assessments indicated profound deficits in conceptual, social, and practical domains, although executive functioning, as reported by caregivers, appeared preserved within a structured environment. This study highlights novel findings regarding phonological discrimination deficits and impaired facial emotion recognition in PMS, aspects that have been underexplored in previous research.

## Introduction

Phelan-McDermid syndrome (PMS) is a neurodevelopmental disorder associated with deletions or mutations on chromosomes 22q13.2 to 22q13.33 and haploinsufficiency of the SHANK3 gene [1,2]. While the phenotype of PMS patients can be variable, several symptoms have been documented: developmental delay and severe language impairment; hypotonia and seizures as neurological symptoms; visual and auditory disturbances, as well as increased pain tolerance perception; and hyperactivity, aggression, and self-injury in behavior [1,2].

The neurocognitive assessment of patients with PMS requires adaptations of traditional testing methods considering profound intellectual and multiple disabilities (PIMD) and language impairment [1]. These adaptations include the use of multiple methods and informants (caregiver and clinician assessments), alternative scoring systems, such as age equivalents, and standardized tools designed for younger populations. Structured clinical observations, caregiver/parental reports, and functional evaluations also contribute to the systematic and clinically meaningful assessments of various domains [1,3]. Although many of these principles are applicable to pediatric neuropsychology, this broad assessment approach is particularly relevant for adult individuals with PMS and rare genetic conditions. In this case report, we analyze an adult, Spanish-speaking woman, genetically confirmed to have PMS due to a 22q13.33 deletion.

## Case presentation

Case description

A 29-year-old woman was referred by the clinic for bipolar disorder to the genetics department for evaluation due to intellectual disability and bipolar disorder. She had no family history of hereditary neuropsychiatry diseases, and her parents were phenotypically normal. She was born at term via cesarean section without complications.

At two years and six months, a language developmental delay was identified, leading to a diagnosis of anaphasic and anarthric language delay. She received therapy until age six. At nine years old, a neuropsychological assessment reported an intelligence quotient (IQ) of 61, with the lowest performance in the language subscale. At the time of the current evaluation, her language is limited to isolated words, short phrases, and communicative gestures.

Hypotonia and gait abnormalities were also diagnosed; at the time of the evaluation, there is no available information regarding tactile or temperature perception deficits; however, her primary caregiver reports a high pain threshold.

She completed basic education with institutional support but discontinued schooling after middle school due to cognitive limitations. The patient has never held formal employment and remains highly dependent on caregivers for daily functioning. Her social interactions are primarily limited to close family members. Behavioral disturbances, including episodes of aggression and repeated attempts to run away from home, have generated significant emotional and psychosocial strain within her family environment.

At the age of 16, she experienced a manic episode marked by irritability, verbal and physical aggression, decreased need for sleep, and increased goal-directed activity, leading to a diagnosis of bipolar disorder. Since diagnosis, the patient has remained on pharmacological treatment. Valproic acid was initially prescribed at 200 mg three times daily and later adjusted to 250 mg twice daily, which continues as her current dosage. Risperidone was started at 2 mg once daily at night, was subsequently reduced to 0.5 mg daily, and is currently administered as a 1 mg/mL oral solution at 0.5 mL once daily. This regimen has been associated with clinical stability. The duration of pharmacological treatment has now extended to approximately 15 years.

**Figure 1:**
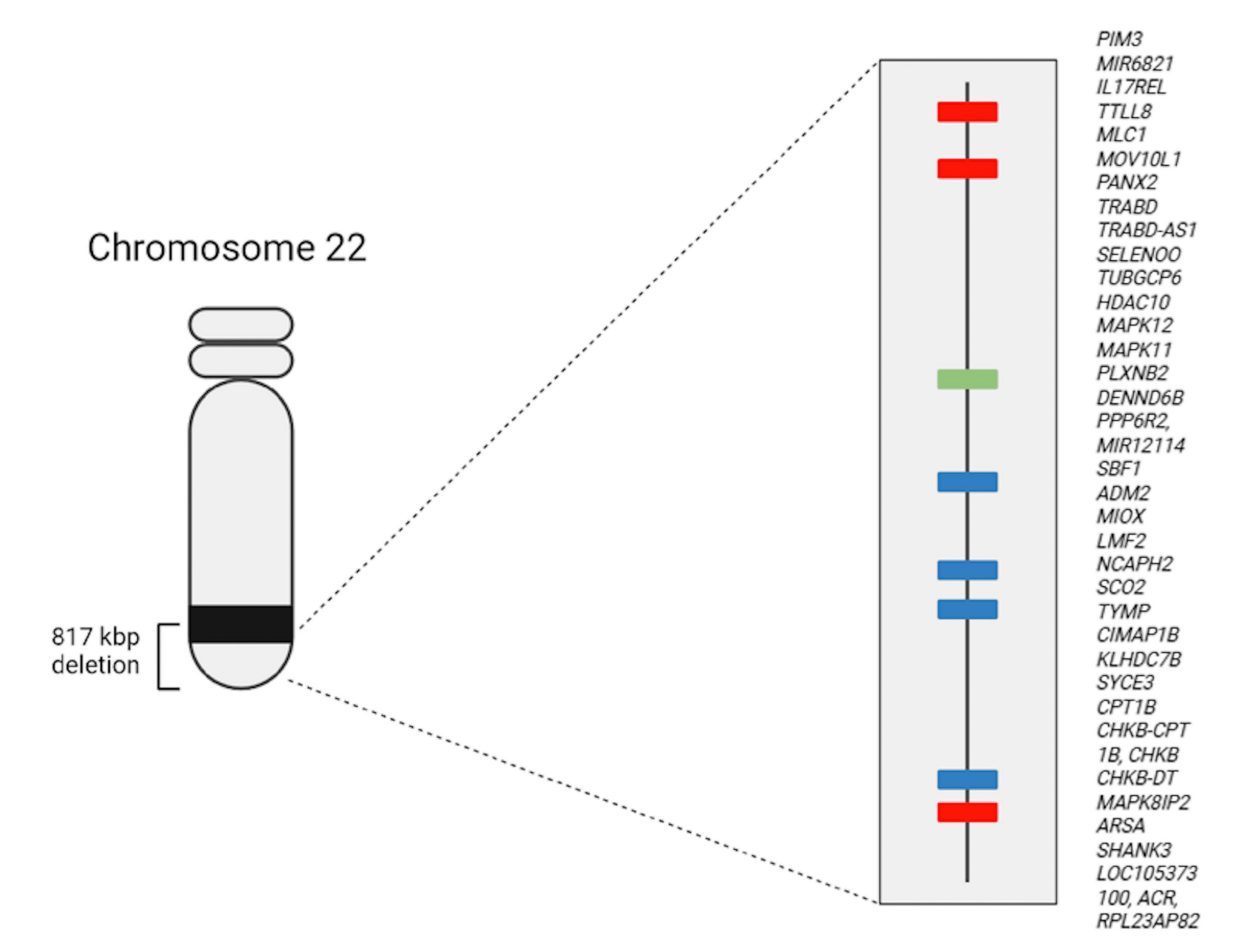
Chromosome 22 ideogram Chromosome 22 ideogram showing the 22q13.33 deletion. The deleted region encompasses 28 genes, most notably SHANK3, whose haploinsufficiency critically contributes to altered neurodevelopmental pathways and the manifestation of the patient's clinical phenotype.

Given her combined neurodevelopmental and psychiatric presentation, genetic testing was conducted. Conventional karyotyping revealed a ring chromosome 22 [46,XX,r(22)(p11q13)], indicating a terminal chromosomal rearrangement. High-resolution DNA microarray analysis (CytoScan 750K, Thermo Fisher Scientific, Waltham, MA USA) further identified an 817 kbp deletion at 22q13.33 [arr(hg38) 22q13.33(49942377_50759338)x1], encompassing 28 Online Mendelian Inheritance in Man (OMIM)-listed genes. Among these is SHANK3, a gene critical for synaptic integrity and neurodevelopment. Haploinsufficiency of SHANK3 and other genes in this region has been strongly associated with the phenotypic spectrum of PMS (Figure 1), thereby confirming the diagnosis in this patient [4].

Cognitive assessment

The cognitive assessment was conducted by a psychologist with a master's degree in clinical neuropsychology, and the supervision of the case and selection of the tests were done by a clinical psychologist with a doctoral degree in psychology. All tests were conducted in Spanish language, and these tests have their Spanish translation and are referred to below:

Peabody Picture Vocabulary Test-III (PPVT-III)

This measures receptive vocabulary and screens verbal ability. It provides age-equivalent data and a proxy for general cognitive ability. The test consists of 16 sets of 12 items, each with a target word and three distractors. The examiner says the word aloud and the examinee selects the corresponding picture. Each item is scored as 0 (error) or 1 (success). IQ, stanines, and age-equivalent scores are available for individuals aged 2.5-90 years. Average IQ scores range from 85 to 115 (mean = 100; standard deviation = 15) [5].

Battelle Developmental Inventory (BDI)

This inventory contains 341 items grouped into the domains of Personal/Social, Adaptive, Motor (Gross and Fine), Communication (Receptive and Expressive), and Cognitive. Results are expressed as Developmental Age Equivalents for each domain. A three-point scoring system is used, where 0 indicates a poor approximation of the requested behavior, 1 indicates an attempt without fully meeting the established criterion, and 2 indicates a response that meets the established criterion. Scores are expressed as z-scores and equivalent ages [6].

Behavior Rating Inventory of Executive Function-Adult Version (BRIEF-A)

This is a caregiver-completed inventory that evaluates everyday executive functioning in adults aged 18-90 years. It consists of 75 items across nine subscales: Inhibit, Shift, Emotional Control, Self-Monitor, Initiate, Working Memory, Plan/Organize, Task Monitor, and Organization of Materials. The inventory uses a Likert scale with three options: N for behaviors that have never been a problem, S for behaviors that have sometimes been a problem, and O for behaviors that are often problematic. It provides results in T-scores, where scores above 65 indicate executive difficulties (mean = 50; standard deviation = 10) [7].

Adaptive Behavior Assessment System-II (ABAS-II)

This is a caregiver-reported questionnaire assessing 11 functional areas, including Motor Skills, Communication, Use of Community Resources, Functional Pre-academic Skills, Daily Living, Health and Safety, Leisure, Self-Help, Self-Direction, Social Skills, and Work. It uses a Likert-type scale with four options, not able, never when necessary, sometimes when necessary, and always when necessary, scored from 0 to 4. It provides raw scores ranging from 0 to 81 in each area. Age-differentiated scales are offered. Scores for the individual areas are expressed as scaled scores (mean = 10; standard deviation = 3), and scores for adaptive domains and the General Adaptive Behavior Index are expressed as IQ scores (mean = 100; standard deviation = 15) [8].

Programa Integrado de Exploración Neuropsicológica Test Barcelona-2 (PIEN-2)

This includes subtests for expressive language (repetition of Syllables, Syllable Pairs, Pseudowords, Words, and Phrases) and tactile perception (Graphesthesia, Morphognosia, and Finger Gnosis). Each item is scored as 0 or 1, with total scores ranging from 0 to 10. Percentile scores are adjusted for the patient's chronological age and education level [9].

Ekman and Friesen Adapted Test

This assesses emotion recognition. Participants view six faces on a computer screen displaying five emotions (Joy, Anger, Fear, Sadness, and Neutral) and identify the corresponding pictogram. Correct identifications score 1 point, while errors score 0 [10].

Results

In Tables 1-6, we describe the scores obtained in receptive vocabulary and verbal ability scales, developmental level, executive functioning, adaptive behavior, expressive language, tactile perception, and emotion recognition task.

**Table 1:** Cognitive assessment: PPVT-III PPVT-III: Peabody Picture Vocabulary Test-III; IQ: intelligence quotient

Test	Raw scores	Normative scores: percentile	Age equivalent	Interpretation
PPVT-III	46	1	4 years and 8 months	IQ proxy: 55 very low

**Table 2:** Cognitive assessment: BDI BDI: Battelle Developmental Inventory

BDI	Raw scores	Standard deviation	Months	Interpretation
Personal/Social	36	2	57-60	Severe impairment
Adaptive	34	-2	61-65	Severe impairment
Motor Skill	35	-2	64-67	Severe impairment
Gross Motor	15	-2	51-60	Severe impairment
Fine Motor	20	-2	60-75	Severe impairment
Communication	23	-2	43-44	Severe impairment
Receptive	12	-2	48-52	Severe impairment
Expressive	11	-2	38-42	Severe impairment
Cognitive	24	-2	46-48	Severe impairment

**Table 3:** Cognitive assessment: BRIEF-A BRIEF-A: Behavior Rating Inventory of Executive Function-Adult Version

BRIEF-A	Raw scores	Normative scores: T-scores	Interpretation
Inhibit	15	57	Normal
Shift	12	60	Normal
Emotional Control	20	60	Normal
Self-Monitor	10	54	Normal
Initiate	13	53	Normal
Working Memory	13	53	Normal
Plan/Organize	17	60	Normal
Task Monitor	11	59	Normal
Organization of Material	16	58	Normal

**Table 4:** Cognitive assessment: ABAS-II ABAS-II: Adaptive Behavior Assessment System-II; Ts: typical score with mean 100 and standard deviation; Sc: scalar score with mean = 10 and standard deviation = 3; GAC: General Adaptive Composite

ABAS-II	Raw scores	Normative scores: Ts and Sc	Interpretation
GAC	-	Ts: 58	Very low
Conceptual	-	Ts: 53	Very low
Social	-	Ts: 65	Very low
Practical	-	Ts: 58	Very low
Communication	49	Sc: 1	Very low
Functional Academics	6	Sc: 1	Very low
Self-Direction	42	Sc: 1	Very low
Leisure Skills	34	Sc: 1	Very low
Social Interaction	56	Sc: 5	Medium
Self-Care	70	Sc: 2	Very low
Home or School Living	42	Sc: 4	Low
Community Use	23	Sc: 1	Very low
Health and Safety	42	Sc: 1	Very low
Work	35	Sc: 1	Very low

**Table 5:** Cognitive assessment: PIEN-2 PIEN-2: Programa Integrado de Exploración Neuropsicológica Test Barcelona-2

PIEN-2	Raw scores	Normative scores: percentile	Interpretation
Expressive language			
Syllables	3	9	Severe impairment
Syllable Pairs	0	1	Severe impairment
Pseudowords	0	1	Severe impairment
Words	3	3	Severe impairment
Phrases (Words)	11	3	Severe impairment
Tactile perception			
Graphesthesia Right	3	3	Severe impairment
Graphesthesia Left	5	5	Severe impairment
Morphognosia Right	6	5	Severe impairment
Morphognosia Left	5	5	Severe impairment
Finger Gnosis Right	4	3	Severe impairment
Finger Gnosis Left	3	3	Severe impairment

**Table 6:** Cognitive assessment: Ekman and Friesen Adapted Test

Ekman and Friesen Adapted Test	Hit	Error
Joy	5	1
Anger	5	1
Fear	1	5
Sadness	1	5
Neutral	0	6

The patient shows a generalized developmental delay and severe impairments in expressive language and comprehension. Adaptive behavior skills in the conceptual, social, and practical domains also yielded very low scores.

Strikingly, the ecological description of executive functions assessed through the BRIEF-A, as reported by the patient's primary caregiver, falls within the normal range. This finding suggests that, within highly structured and caregiver-adapted contexts, the patient can organize behavioral plans and demonstrates adequate performance in inhibition, cognitive flexibility (shift), self-monitoring, and emotional control.

Specific assessment of expressive language indicates that the patient is more successful in reproducing syllables, words, and short phrases compared to reproducing syllable pairs and pseudowords. This suggests that phonetic discrimination is significantly impaired in this case. Additionally, poor performance was observed in the recognition of tactile perception in both the right and left hands for shapes and figures, as well as difficulties in finger gnosis.

Regarding a subdomain of social cognition, the patient showed facilitated recognition of emotional facial expressions, particularly joy and anger. However, expressions such as fear and sadness and especially neutral expressions were difficult for the patient to identify.

The patient continues to receive attention from psychiatric genetics and general psychiatry services.

## Discussion

PMS is a rare genetic condition that can be challenging to assess due to multiple disabilities and severe language impairment. It is documented that adults with PMS can have significant difficulties in communication, social interaction, autonomy, and cognitive skills caused primarily by pervasive developmental delay and, subsequently, by cognitive decline observed in the follow-up until middle age is reached [1-3,11].

In this case report, the neuropsychological assessment points to severe impairments in language production with no structural abnormalities in the bucco-phonatory apparatus. Language is constructed with isolated but articulated words and communicative gestures, such as "yes", "no", "hello", and "bye". This reflects adequate skills to use symbolic communication, consistent with findings in pediatric [12] and adult PMS populations [13].

Additionally, the patient demonstrated accurate repetition of syllables and words, but severe difficulties with syllable pairs and pseudowords. These findings are consistent with the work of Brignell et al., who performed the most comprehensive assessment of language profiles of PMS patients [12]. The authors noted that verbal individuals with PMS can precisely perform consonant and vowel repetitions in 74-100% of the cases. However, phoneme repetition errors are observed in 50% of items. In our study, the patient was able to repeat isolated phonemes more accurately; however, when asked to perform a deeper analysis to distinguish between similar phonemic units or in complex stimuli such as pseudowords, she failed in all items. This highlights a specific difficulty in phoneme discrimination, which provides a novel contribution to the understanding of language impairments in PMS. Our patient's language profile, expressive language with the reproduction of simple words and phrases, difficulties in phonological processing, better performance in language comprehension (in our case study, she obtained an age equivalent of 4.8 according to the Peabody instrument for concept knowledge and verbal abstraction), and the use of transitive gestures or communication gestures, is consistent with the literature [11-13].

Deletions at 22q13.33 have been associated with atypical sensory responses, including hypo- or hyper-reactivity to sensory stimuli [14,15]. Specifically, altered tactile perception, temperature, touch, and pain sensitivity have been reported in 70% of cases [15]. In this study, we observed specific impairments in graphesthesia, morphognosia, and finger gnosis. These tasks require the representation and integration of tactile stimuli and spatial localization skills. A series of cases described in 2008 (14) describes similar findings, reporting tactile and finger gnosis deficits of up to 2 standard deviations in eight studied cases. Such impairments align with animal models: Shank3b-mutant mice exhibit reduced texture discrimination ability accompanied by hippocampo-S1 hypoconnectivity and diminished cortico-hippocampal circuit activation [16].

Several descriptions of phenotypic features of PMS show us the variability in the parameters of severity of intellectual disability, language impairment, or neurological symptoms as described above [3,11-13]; this implies that these clinical features, even with severe disturbances, can be considered as a continuum. This same variability could be found in other domains such as autism spectrum characteristics reported in these patients. The most recent descriptions of autism spectrum traits in several genetic syndromes suggest that more than 50% of the evaluated individuals with PMS present difficulties with eye contact, social smiling, and positive social responses [17]. Egger et al. [18] described cognitive alexithymia difficulties in identifying, verbalizing, and analyzing emotions, in a population of seven adults with PMS who were tested with the Bermond-Vorst Alexithymia Questionnaire and the Toronto Alexithymia Scale. The patient displays a specific deficit in the recognition of facial emotions, such as fear, sadness, or neutral faces, but a better performance pointing out the pictogram that was associated with happy and angry faces. Social cue perception is considered an essential component of social cognition, and it has been shown that patients with neurodevelopmental disorders often show difficulties in identifying emotional expressions in others, especially emotions such as surprise or fear [18,19]. We consider that the description of failures in the recognition of facial emotions is a novel contribution to the study of PMS.

The BRIEF-A scale answered by the caregiver indicates that the patient does not show significant difficulties in the executive functions of daily life. It is important to point out that the environment in which this behavior is evaluated has been highly structured by the caregiver, which favors the performance of routines and activities. However, lower scores were obtained in the traditional executive function test [9,12,17]. In the same line, the assessment of adaptive behavior performed with ABAS-II showed similar parameters to those reported in the literature regarding the conceptual, social, and practical skills, which are considered impairment [3].

## Conclusions

In this case report, we propose a neuropsychological battery to assess the neurocognitive characteristics of Spanish-speaking PMS adult patients.

One of the main contributions of this proposal is the study of a specific aspect of language impairment that affects both comprehension and expression: phonological discrimination. Through an assessment that is simple to administer and score, clinicians can identify whether a difficulty in correctly identifying the phonemes that make up words is present. This level of precision moves us away from categorical statements such as "presence" or "absence" of language impairment and instead opens a future line of research into the specific mechanisms underlying this deficit and, potentially, targeted interventions. Along similar lines, although tactile perception deficits have been documented in individuals with PMS, our report offers a possible option for identifying the severity of this impairment.

Perhaps one of the most important reflections illustrated by this case report is that, while individuals with PMS may present with severe executive functioning impairments when assessed using traditional instruments, ecological evaluations of these same functions may yield different outcomes. In this case, a stimulus-controlled environment, the learning of specific behavioral routines, and caregiver support contributed to better functioning in daily life activities. As clinicians, it is essential to consider the patient's functioning within their real-life context, rather than drawing conclusions about low functioning based solely on traditional executive function assessments.

We propose that this evaluation approach may complement existing assessments of developmental level, adaptive behavior, and intellectual disability in this population. Future directions in this field could include additional tests within the domain of social cognition, such as the identification of social cues in prosody.
